# The impact of social media information exposure on appearance anxiety in young acne patients: a moderated chain mediation model

**DOI:** 10.3389/fpsyg.2024.1409980

**Published:** 2024-08-02

**Authors:** Zhijian Zhang, Min Zhou

**Affiliations:** College of Journalism and Communication, Huaqiao University, Xiamen, China

**Keywords:** young acne patients, social media information exposure, internalization of beauty ideals, fear of negative evaluation, appearance anxiety, self-esteem, a moderated chain mediation model

## Abstract

**Introduction:**

The boom of social media has provided a wider space for ordinary people to display themselves, but visual presentation has also intensified the focus on appearance, which in turn triggers anxiety about appearance. The study aims to investigate the impact of social media information exposure on appearance anxiety in young acne patients and the pathways and mechanisms that cause this effect.

**Methods:**

A moderated chain mediation model was constructed, and a questionnaire was used to collect information on social media information exposure, internalization of beauty ideals, fear of negative evaluation, self-esteem, and appearance anxiety in young acne patients (*N* = 382), and the relationships between the variables were explored through regression analysis.

**Results:**

The results show that there was a significant path of effect (*t* > 2.5, *p* < 0.05) between social media information exposure, internalization of beauty ideals, fear of negative evaluation, and appearance anxiety. Self-esteem significantly moderated the relationship between social media information exposure and internalization of beauty ideals (*t* < −2, *p* < 0.05).

**Discussion:**

In conclusion, in young acne patients, internalization of beauty ideals and fear of negative evaluation chain mediated the association between social media information exposure and appearance anxiety, and young acne patients’ internalization of beauty ideals was inversely correlated with their level of self-esteem.

## 1 Introduction

In recent years, topics such as “palm-sized face,” “A4 waist,” “vegan beauty,” “comic legs,” “swan neck” and other related topics have dominated various social media hot lists. This has had a significant impact on young people’s body image, exacerbated their anxiety about their appearance, and led many beauty lovers to use plastic surgery, strict diets, and other extreme measures to make up for their shortcomings (Fardouly et al., 2017; McComb and Mills, 2022; Wang et al., 2024).

The phenomenon of appearance anxiety is particularly common in the youth population. According to a study by Capital Campus Press Union, 59.03% of university students had some degree of appearance anxiety, with a higher proportion of female students (59.67%) than male students (37.14%) having moderate anxiety (China Youth Daily, 2021). Studies have shown that acne has a worldwide prevalence of 9.4%, making it the eighth most prevalent disease in the world (Tan and Bhate, 2015). The prevalence of acne in China is 8.1%, with 74.3% of adolescents suffering from acne (Zhang et al., 2012).

Acne is a common inflammatory skin disease, with the highest incidence occurring in 14–19 ages. And girls usually develop acne earlier than boys. In recent years, persistent acne has become increasingly common in adult women (Dréno et al., 2020). Topical medication is the most common and basic treatment for acne, mild and mild-to-moderate acne can be treated with topical medication. Severe acne can all be treated with topical medication supplemented with systemic therapy. Physical and chemical therapeutic modalities, like lasers and red-blue light, are also useful adjunctive measures for treating acne, but rarely involve the psychological treatment of patients with acne (Thiboutot et al., 2009; Keyal et al., 2016; Bhargava et al., 2018).

Current research suggests that acne sufferers are susceptible to mental health effects due to acne problems (Bewley et al., 2016). In contrast to clinical indications like pain and bleeding, psychological issues are frequently the most common morbidity expression of acne disease (Koo, 1995). Acne sufferers may also be more neurological sensitivity, introverted, and anxious in social circumstances than usual (Varade et al., 2016). In studies of acne in adolescents, it has been found that the negative effects of acne on adolescents are significant, and they can greatly reduce their self-esteem due to dissatisfaction with their appearance (Hedden et al., 2008). It has also been found that stress leads to immune dysfunction and it is a cause of many diseases (Dhabhar, 2009). Some studies have already confirmed that stress is an important factor in the onset, exacerbation, and recurrence of many skin diseases (Dixon et al., 2018), and the emotional stress aggravates acne to a great extent (Aktan et al., 2000).

Currently, some studies have explored the issue of social media information exposure and appearance anxiety (Walker et al., 2015; Hawes et al., 2020; Prasad et al., 2023), but few studies have examined the issues related to young acne patients, and even fewer have explored the intrinsic influence mechanisms and pathways. Therefore, this study takes young acne patients as the main research group to explore the influence path and formation mechanism of social media information exposure on acne patients’ appearance anxiety, which has certain reference value for clinical medicine to provide better treatment for acne patients.

From the perspective of social psychology, the problem of appearance anxiety triggers many adverse emotions, causing serious negative impacts on academics, careers, and socialization, and even resorting to extreme methods to make up for the shortcomings of appearance (Phillips et al., 2004; Kollei et al., 2012). China Consumers’ Association pointed out that in recent years, the medical beauty industry has continued to grow in popularity, and the influence of negative social culture such as “appearance anxiety” has deepened, so that medical beauty is no longer just a popular choice for “mature” people, but more and more minors are also deeply involved in it (China Consumers’ Association, 2023).

According to the “2022 White Paper on the Medical Beauty Industry,” the age of medical beauty consumers continues to decrease, with 11.12% of consumers under the age of 20 (2022 White Paper on the Medical Beauty Industry, 2022), which has also led to a continuous increase in the number of medical beauty complaints in recent years, and the order of complaints in the field of events is always in the top ten (Li and Li, 2022).

Therefore, this study can make more people aware of the psychological impact of social media information exposure on young acne patients. Appearance anxiety is not just a simple “beauty problem,” and the whole society should pay more attention to the problem of young acne patients’ appearance anxiety, and takes certain measures to help young people develop physical and mental well.

### 1.1 Relationship between social media information exposure and appearance anxiety

The effects of social media exposure on body intentions have been a hot topic of research in the field of psychology in recent years. According to some studies, social media can have a lot of positive effects, especially for young people. It can not only satisfy their entertainment needs, but also share behaviors such as selfies to satisfy their needs for self-presentation, thus promoting social interactions among adolescents, enhancing their sense of belonging, and obtaining more social capital (Boursier et al., 2020). However, there are also many studies that indicate that exposure to social media information can exacerbate dissatisfaction with appearance and produce appearance anxiety.

According to Tanya Hawes, who says that social media reinforces the public’s preoccupation with appearance and brings with it emotional problems such as depression, social anxiety, and appearance anxiety (Hawes et al., 2020). After reviewing 77 pertinent studies, Prasad Sakshi et al. discovered that various social media usage patterns, including passive use, task processing, and emotional investment, can cause various mental health issues in youths (Prasad et al., 2023). Additionally, Morgan Walker et al. came to the conclusion through an online survey that activities related to appearance comparison and statements about obesity on the Internet can lead to serious eating disorders (Walker et al., 2015). Accordingly, Hypothesis 1 is put out in this paper: Social media information exposure is significantly and positively related to appearance anxiety in young acne patients.

### 1.2 The mediating role of internalization of beauty ideals

Internalization of beauty ideals is the adoption of the ideal appearance standards in one’s social environment, in terms of actions and values (Stice et al., 2000). After exposing female students in grades 7 and 9 to idealized images, Sarah J. Durkin and Susan J. Paxton measured the participants’ levels of body satisfaction, depression, anxiety, and anger. They discovered that there was a significant decline in body satisfaction and a significant rise in depressive states (Durkin and Paxton, 2002). Women who watched videos portraying an ideal appearance decreased in satisfaction with their appearance and eventually internalized this ideal appearance state, as demonstrated empirically by Jade C. Gurtala and Jasmine Fardouly (Gurtala and Fardouly, 2023). Using a controlled experimental design, McComb, SE and Mills, JS discovered that participants who were exposed to the media’s ideal body were much less satisfied with their weight and looks than the control group (McComb and Mills, 2022). Accordingly, Hypothesis 2 is put out in this paper: In young acne patients, internalization of beauty ideals plays a major mediating role between social media information exposure and appearance anxiety.

### 1.3 The mediating role of fear of negative evaluation

The worry and fear of receiving a poor evaluation that people feel in social settings is known as fear of negative evaluation (Carleton et al., 2011). Research by Sophie E. Carruthers et al. revealed that when using Facebook, individuals with social anxiety experienced increasing levels of anxiety and negative thoughts (Carruthers et al., 2019). Myeongki Jeong et al. discovered that users’ discomfort increased over time as a result of being exposed to opposing viewpoints more frequently when using social media (Jeong et al., 2019). Meanwhile, Reilly et al., 2018 noted a significant positive relationship between repetitive negative thinking and social appearance anxiety. Accordingly, Hypothesis 3 is put out in this paper: In young acne patients, fear of negative evaluation plays a major mediating role between social media information exposure and appearance anxiety.

### 1.4 The chain mediating role of internalization of beauty ideals and fear of negative evaluation

Individuals with a high degree of internalization of beauty ideals are more likely to take the perfect image that is created by the social environment as their appearance goal, and these people will amplify their flaws, then generate false concerns about perfectionism - viewing small errors as overall failures (Hewitt and Flett, 1993; Stoeber and Yang, 2015). The majority of researches have found that there are adaptive and non-adaptive components to perfectionism (Dunkley et al., 2000; Cox et al., 2002). People with adaptive perfectionism show positive psychological states, seting high personal standards for themselves, and they do not worry about excessively mistakes, doubt actions, self-criticize, they are also not afraid of failing to live up to their own standards and the high expectations of others. However, non-adaptive perfectionists frequently feel that other people have high standards for them and will exaggerate the differences between their own and other people’s assessments, which increases their anxiety of receiving negative evaluations (Stoeber and Otto, 2006; Yap et al., 2016). In summary, those with a high degree of ideal beauty internalization are likely to be non-adaptive perfectionists.

Meanwhile, it has been demonstrated that a misguided concern for perfectionism will make college students feel more anxious about their appearance and more dissatisfied with it (Hewitt and Flett, 1993; Stoeber and Yang, 2015). According to Pawijit et al. (2019), there is a strong positive correlation between body image dissatisfaction and fear of negative evaluation, which in turn causes social appearance anxiety. Accordingly, Hypothesis 4 is put out in this paper: There is a chain mediating role of internalization of beauty ideals and fear of negative evaluation between social media information exposure and appearance anxiety in young acne patients.

### 1.5 The moderating role of self-esteem

Studies have pointed out that low self-esteem individuals are more likely to use online interactions to compensate for offline interactions, and even develop social media addictions (Busalim et al., 2019). Abdollahi and Abu Talib (2016) noted that people with low physical self-esteem are more inclined to think negatively about themselves, resulting in anxiety and tension. Therefore, individuals with low self-esteem may be more susceptible to “beauty ideals” messages in social media and may internalize beauty ideals to a greater extent, affecting their physical and mental health in the process of negative evaluation. Accordingly, Hypothesis 5 is put out in this paper: Self-esteem moderates the relationship between social media information exposure and internalization of beauty ideals in young acne patients.

## 2 Methodology

### 2.1 Research methodology

Although this paper has a certain color of clinical medical research, but appearance anxiety belongs to a typical social psychological state and young acne patients are a unique social psychological research object with a sizable group. So this study belongs to the research category of social psychology. At the same time, considering that most of the social psychology studies similar to the topic of this study use the questionnaire method (Sumter et al., 2018; Cho et al., 2019; Pan and Mu, 2024), the questionnaire method is used as the main research method of this paper, and the regression analysis of the data is carried out by using the SPASS software with the PROCESS macro program.

### 2.2 Objects of study

This study focused on young acne patients. According to the “Medium and Long term Youth Development Plan (2016–2025)” announced by the State Council of China in April 2017, the age range of young people is 14–35 years old (Medium and Long-term Youth Development Plan (2016-2025), 2017). Therefore, the subjects in this study were young people from age 14 to 35 and they conformed to the acne grades classified in the Chinese Acne Treatment Guidelines: mild (grade I) manifested by pimples only; moderate (grade II) manifested by the presence of inflammatory papules; moderate (grade III) by the presence of pustules; and severe (grade IV) by the presence of nodules and cysts (Ju, 2019).

The questionnaire design was based on established and mature scales, adapted accordingly to the Chinese context. A previous survey was conducted, and the previous survey results indicated that the present study scale applies to the study of the effects of social media information exposure on appearance anxiety in young Chinese acne patients. The questionnaire for this study was created using Questionnaire Star (the biggest and oldest online questionnaire platform in China). It was distributed over WeChat, Weibo, and other social media platforms which are the most influential in China. A total of 544 questionnaires were collected, with 382 valid questionnaires and a 70.2% recovery rate. Among them, 94 (24.6%) were young male acne patients and 288 (75.4%) were young female acne patients; 259 (67.8%) were young acne patients between the ages of 14 and 25, and 123 (32.2%) were acne patients between the ages of 26 and 35.

### 2.3 Measuring tools

#### 2.3.1 Social media information exposure scale

Using the social media information exposure scale developed by Mars et al. (2015). The scale evaluates young acne patients’ use of social media from both active and passive questioning perspectives. The questions are scored on the five-point Likert scale, with higher scores indicating more significant social media information exposure in patients. The Cronbach’s α for this scale in this study was 0.872.

#### 2.3.2 Internalization of beauty ideals scale

Using the third edition of the sociocultural attitudes toward appearance scale by Thompson (Thompson et al., 2004). The scale was divided into 3 questions to measure the internalization of beauty ideals in young acne patients in terms of general internalization and stress dimensions. The questions were based on the five-point Likert scale, with higher scores indicating a higher degree of internalization of beauty ideals in the patients. The Cronbach’s α for this scale in this study was 0.762.

#### 2.3.3 Fear of negative evaluation scale

Using the brief version of the fear of negative evaluation scale by Leary (1983). The original scale was set up to ask both forward and reverse questions, and research has shown that scales that ask questions in a single forward dimension have higher reliability and validity (Lin, 2016). Therefore, forward questioning was used in this study. The scale consisted of five questions, all of which were scored on the five-point Likert scale, with higher scores indicating a higher level of fear of negative evaluation in the patients. The Cronbach’s α for this scale in this study was 0.874.

#### 2.3.4 Appearance anxiety scale

Using the social appearance anxiety scale developed by Hart et al. (2008). The scale consists of six questions and is assigned a score using a five-point Likert scale, with higher scores indicating a more severe case of appearance anxiety in young acne patients. The Cronbach’s α for this scale in this study was 0.885.

#### 2.3.5 Self-esteem scale

Using the self-esteem scale developed by Winch and Rosenberg (1965). It was used to assess the patients’ feelings of self-worth and self-acceptance. The scale was designed with three reverse questions, all on the five-point Likert scale. The questions were reverse scored using SPSS software, a higher score indicated a higher level of self-esteem in the patient. The Cronbach’s α for this scale in this study was 0.843.

## 3 Results

### 3.1 Model measurement

Before analyzing the data, a validation factor analysis was conducted to test the fit of the model. The results showed that the structural validity (X2/df = 2.23, RMSEA = 0.057, GFI = 0.923, CFI = 0.955, IFI = 0.955, and TLI = 0.946), combined validity (CR > 0.7 and AVE > 0.5), and discriminant validity (Table 1) of the model reached the desirable indexes, which indicated that the model could observe the data well.

**Table 1:** Combined and discriminant validity tests.

Variables	CR	AVE	Social media information exposure	Internalization of beauty ideals	Fear of negative evaluation	Appearance anxiety	Self-esteem
Social media information exposure	0.877	0.781	0.884				
Internalization of beauty ideals	0.797	0.582	0.383	0.763			
Fear of negative evaluation	0.876	0.586	0.322	0.444	0.765		
Appearance anxiety	0.886	0.566	0.401	0.415	0.751	0.752	
Self-esteem	0.849	0.656	−0.068	−0.185	−0.400	−0.466	0.810

Next, we conducted a one-factor validation factor analysis. The results showed that the fit indices of the model (X2/df = 10.093, RMSEA = 0.154, GFI = 0.696, CFI = 0.643, IFI = 0.645, and TLI = 0.598) were significantly different from those of the original model, suggesting that there was no obvious common method bias in the measurement of the original model.

### 3.2 Correlation analysis between variables

As shown in Table 2, social media information exposure was significantly and positively correlated with internalization of beauty ideals, fear of negative evaluation, and appearance anxiety (*r* > 0.28, *p* < 0.01); internalization of beauty ideals was significantly and positively correlated with fear of negative evaluation and appearance anxiety (*r* > 0.45, *p* < 0.01); fear of negative evaluation was significantly and positively correlated with appearance anxiety (*r* > 0.66, *p* < 0.01); and self-esteem was significantly and negatively correlated with internalization of beauty ideals, fear of negative evaluation, and appearance anxiety (|*r*| > 0.18, *p* < 0.01).

**Table 2:** Results of descriptive statistics and correlation analysis.

Variables	M	SD	Social media information exposure	Internalization of beauty ideals	Fear of negative evaluation	Appearance anxiety	Self-esteem
Social media information exposure	3.149	0.997	1				
Internalization of beauty ideals	3.310	0.864	0.376**	1			
Fear of negative evaluation	3.623	0.797	0.286**	0.474**	1		
Appearance anxiety	3.403	0.855	0.357**	0.458**	0.668**	1	
Self-esteem	3.345	0.947	−0.058	−0.186**	−0.365**	−0.422**	1

**P < 0.01.

### 3.3 Mediation test

**Figure 1:**
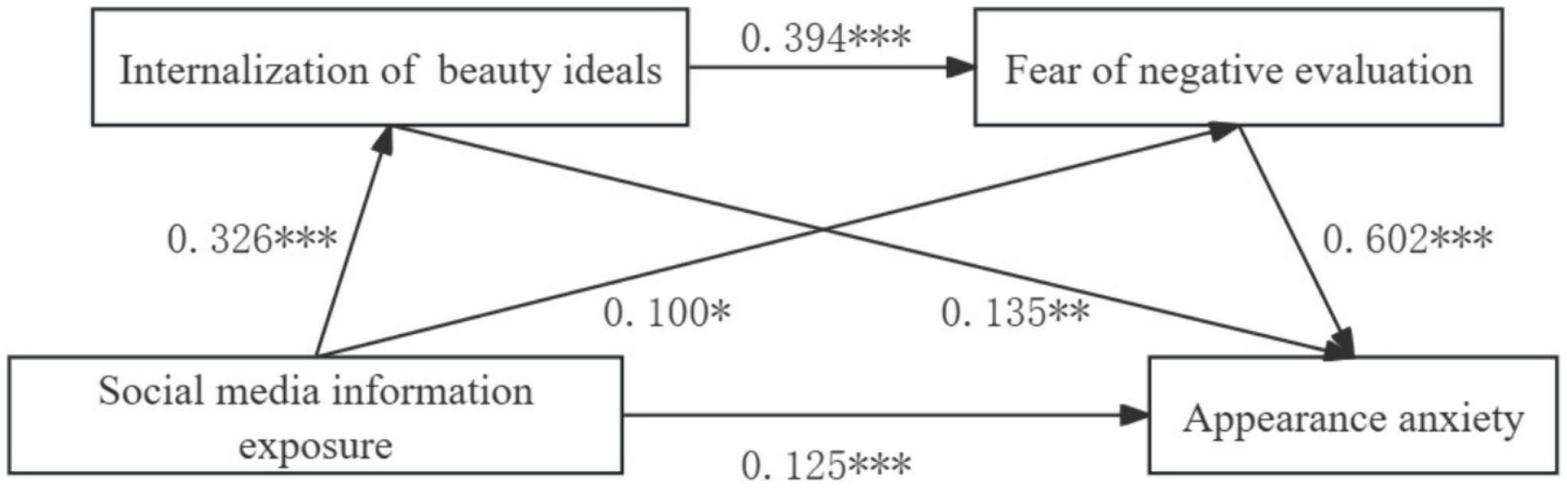
Path diagram of the effect of social media information exposure on appearance anxiety. *p < 0.05; **p < 0.01; ***p < 0.001.

Model 6 in the PROCESS macro program developed by Hayes was used to analyze the mediating role of the variables in the study (Hayes, 2018). The results showed (Table 3 and Figure 1) that social media information exposure significantly and positively predicted appearance anxiety (*β* = 0.125, *p* < 0.001), thus Hypothesis 1 was valid; social media information exposure significantly and positively predicted internalization of beauty ideals (*β* = 0.326, *p* < 0.001), and fear of negative evaluation (*β* = 0.100, *p* < 0.05); internalization of beauty ideals significantly and positively predicted fear of negative evaluation (*β* = 0.394, *p* < 0.001), and appearance anxiety (*β* = 0.135, *p* < 0.01); fear of negative evaluation significantly positively predicted appearance anxiety (*β* = 0.602, *p* < 0.001).

**Table 3:** Regression analysis between variables.

Regression equations		Overall fit indices			Significance of regression coefficient	
Outcome variables	Predictor variables	*R*	*R* ^2^	*F*	*β*	*t*
Internalization of beauty ideals	Social media information exposure	0.376	0.141	62.604	0.326	7.912***
Fear of negative evaluation	Social media information exposure	0.488	0.238	59.271	0.100	2.580*
	Internalization of beauty ideals				0.394	8.831***
Appearance anxiety	Social media information exposure	0.700	0.490	120.834	0.125	3.637***
	Internalization of beauty ideals				0.135	3.137**
	Fear of negative evaluation				0.602	13.337***

*P < 0.05;**P < 0.01;***P < 0.001.

A sample of 5,000 times was taken to test the mediating effects of the study variables using the bias-corrected percentile Bootstrap method. The results showed (Table 4) that the 95% confidence intervals of the three paths did not include “0,” indicating that the mediating effects of the three paths were significant and hypotheses 2, 3, and 4 were valid. The total mediating effect value was 0.181, and the relative mediating effects of the three paths were 24.31, 33.15, and 42.54%, respectively.

**Table 4:** Mediating effects of internalization of beauty ideals and fear of negative evaluation between social media information exposure and appearance anxiety.

Trails	Effect	Relative mediation effect	BootSE	Boot LLCT	Boot ULCT
Total mediating effects: Ind1 + Ind2 + Ind3	0.181		0.030	0.125	0.241
Ind1: social media information exposure - internalization of beauty ideals-appearance anxiety	0.044	24.31%	0.016	0.014	0.078
Ind2: social media information exposure - fear of negative evaluation - appearance anxiety	0.060	33.15%	0.024	0.015	0.106
Ind3: social media information exposure - internalization of beauty ideals - fear of negative evaluation - appearance anxiety	0.077	42.54%	0.015	0.050	0.110

### 3.4 Moderation test

The moderating role of self-esteem between social media information exposure and internalization of beauty ideals was analyzed using model 83 in the PROCESS program developed by Hayes (2018). The results showed (Table 5) that the multiplicative term for social media information exposure and self-esteem significantly and negatively predicted internalization of beauty ideals after the addition of the moderating variable of self-esteem, which indicated a moderating role of self-esteem between social media information exposure and internalization of beauty ideals, and Hypothesis 5 was established.

**Table 5:** Regression results from the moderating role of self-esteem between social media information exposure and internalization of beauty ideals.

Regression equations		Goodness-of-fit indicator			Significance of coefficients	
Outcome variable	Predictor variables	R	*R* ^2^	F	β	t
		0.422	0.178	27.312		
Internalization of beauty ideals	Social media information exposure				0.312	7.696***
	Self-esteem				−0.145	−3.406**
	Social media information exposure × self-esteem				−0.090	−2.105*

*P < 0.05, **P < 0.01, ***P < 0.001.

**Figure 2:**
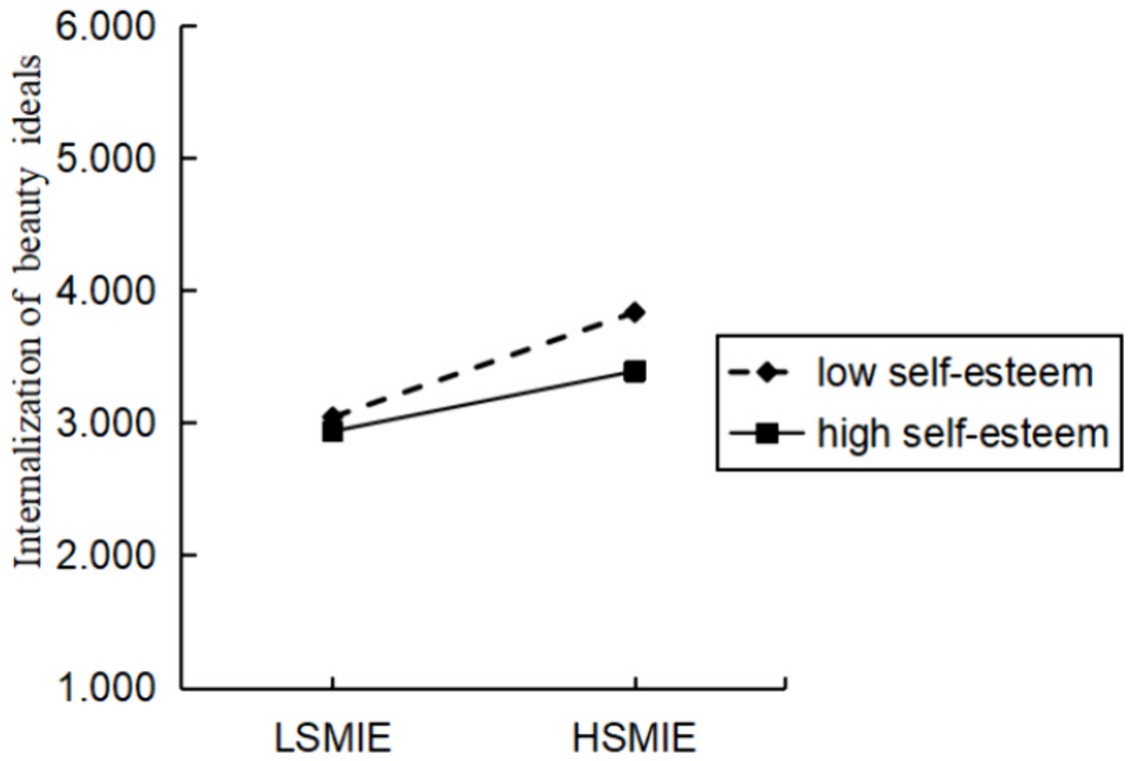
The moderating role of self-esteem between social media information exposure and internalization of beauty ideals. LSMIE, low social media information exposure; HSMIE, high social media information exposure.

Further analysis of simple slopes showed (Figure 2) that when self-esteem levels were higher (one standard deviation above the mean), the regression coefficient (*t* = 3.855, *p* < 0.001) of social media information exposure on the internalization of beauty ideals was lower than when self-esteem levels were lower (one standard deviation below the mean; *t* = 7.179, *p* < 0.001). This suggests that self-esteem attenuates the positive predictive effect of social media information exposure on the internalization of beauty ideals.

## 4 Discussion

With the penetration of social media in daily life, it not only affects people’s daily habits, but also increases the mental burden of the young group and creates many psychological problems (Abi-Jaoude et al., 2020). Appearance anxiety has emerged as a significant psychological issue affecting today’s youth in recent years (China Youth Daily, 2021). Fewer research has examined the pathways and mechanisms by which social media information exposure influences appearance anxiety, despite some studies’ confirmation that using social media lowers the satisfaction of individuals with their looks (Walker et al., 2015; Hawes et al., 2020; Prasad et al., 2023). In addition, acne is the eighth most common disease worldwide, with a high patient population. People with acne are more likely than the general population to experience anxiety states (Darji et al., 2017). However, little study has been done on appearance anxiety in acne patients. As the backbone of a country’s development, exploring the psychological problems of young acne patients is not only helpful to the development of individuals but also important to the progress of the whole society. This paper aims to investigate the impact of social media information exposure on appearance anxiety in young individuals with acne, including its intrinsic influence pathways and mechanisms.

By building a moderated chain mediation model and conducting an empirical analysis of 382 young acne patients, the study finding that social media information exposure exacerbates appearance anxiety in young acne patients, it expands on earlier research findings (Walker et al., 2015; Hawes et al., 2020; Prasad et al., 2023). While some researches point that the existence of different aesthetic perspectives on social media, which encourages more people to be willing to accept themselves and show real themselves which alleviating the phenomenon of look anxiety (Wu et al., 2024). However, more studies have pointed out that exposure to social media can affect their dissatisfaction with appearance, especially when exposed to social media activities regarding appearance comparison and judgment, which can lead to more sensitivity and anxiety about appearance as well as psychological problems such as depression than social media exposure behavior alone (Hawes et al., 2020). This is consistent with the results of this study.

The findings suggest that social media information exposure can reinforce young acne patients’ appearance anxiety through internalization of beauty ideals and fear of negative evaluation alone. Firstly, the fundamental attributes of social media bring commercial value to it, and the presence of advertisers in social media has become a common phenomenon. The ideal image portrayed by advertisers is a presentation of the stereotypical impression of beauty in society and culture, and this perfect and unrepeatable image leads to the difficulty for ordinary audiences to be satisfied with their external image. Over time, they internalize this idealized state of beauty and continuously strengthen the scrutiny of the self in social comparisons, widening the gap between the ideal and real versions of themselves (Christoforou, 2015).

Furthermore, social media serves as a platform for young people to show themselves. They are eager to post selfies online in the hopes of boosting their confidence and self-worth through other people’s approval. This proves that appearance anxiety is closely related to the need to make a positive impression on others (Boursier et al., 2020). This also supports the hypothesis of the present study that the fear of negative evaluations is due to the fear of not getting approval from others for their image appearance during social media information exposure, thus exacerbating their appearance anxiety.

Meanwhile, we found that internalization of beauty ideals and fear of negative evaluation had a chain mediating effect between social media information exposure and appearance anxiety in young acne patients. This implies that the internalization of beauty ideals during social media information exposure reinforced the young acne patients’ fear of negative evaluation, which in turn caused appearance anxiety. Cultivation theory suggests that the mimetic environment constructed by the media subconsciously influences people’s judgment of the objective world, and the longer the exposure to the media, the closer people’s perception of the real world will be to the picture constructed by the media (Gerbner and Gross, 1976). Self-objectification occurs when people embed social values about appearance in social media into their value systems (Calogero et al., 2005), that is, the emphasis is on looking at oneself through a third perspective and focusing primarily on one’s visible attributes (Fredrickson and Roberts, 1997). Those with higher levels of internalization of beauty ideals are more likely to hold themselves to the norms about appearance presented by the media, exacerbating the level of concern about being scrutinized by others, creating a fear of negative evaluation, and ultimately causing appearance anxiety (Hewitt and Flett, 1993; Stoeber and Yang, 2015; Pawijit et al., 2019).

In addition, we found that self-esteem moderated the relationship between social media information exposure and the internalization of beauty ideals in young acne patients. On the one hand, those with low self-esteem were more likely to be addicted to social media, and thus they were more likely to receive messages about “beauty ideals” (Busalim et al., 2019); On the other hand, low self-esteem individuals make more frequent and extreme upward social comparisons during social media information exposure (Midgley et al., 2021). Therefore, low self-esteem acne groups are more inclined to compare their appearance to the beauty ideals, and the more they internalize the beauty ideals.

## 5 Conclusion

### 5.1 General conclusions

The results of this study concluded that young acne patients with greater exposure to social media information have a higher degree of internalization of ideal beauty and are more likely to be fearful of negative evaluations, which can exacerbate their appearance anxiety. Additionally, it was found that young acne sufferers with higher self-esteem had lower levels of internalization of ideal beauty.

In conclusion, social media’s development has both advantages and disadvantages. On the one hand, it gives more space for ordinary people to display themselves, but at the same time, it also contributes to the growth of the face value economy, exacerbates the scrutiny of appearance in society as a whole and reinforces the general audience’s feelings of inferiority about their appearance. Therefore, not only do we need to call on young acne patients to accept themselves, but all sectors of society need to pay more attention to the mental health of acne patients. First, studies have shown that the poor psychological state of acne patients can exacerbate the severity of their acne (Dhabhar, 2009; Dixon et al., 2018), so the medical community should take the psychological problems of acne patients as an important consideration in the process of acne treatment; secondly, public health departments should strengthen attention to psychological problems and take effective measures to relieve the appearance anxiety of young acne patients; finally, media practitioners need to create a pluralistic and healthy aesthetic pattern to help alleviate the public’s anxiety about their appearance and improve their mental health.

### 5.2 Limitations and prospects

There are still some limitations in this paper that should be added and expanded in the future. First, because the main purpose of this study was to examine the relationship between social media information exposure and variables such as appearance anxiety and internalization of ideal beauty etc. And with the limitation of research resources, so this study investigated the psychosocial status of young acne patients as a whole without further subdividing the severity of their acne specifically. Second, although this study belongs to the field of social psychology research, and it is reasonable and feasible to use the questionnaire method to conduct the study, the inability to accurately assess the responses of the research subjects is also one of the inevitable drawbacks of the questionnaire method, so in the future, we can try to make up for this problem through other methods. Third, all the samples in this paper were from young acne patients in China, so the findings may not apply to other countries with different cultural backgrounds; Fourth, this paper is more generalized in the investigation of the information contacted by young acne patients on social media, and subsequent studies can be more specific subdivided when examining this part. Finally, this paper only examined the mediating role of internalization of beauty ideals and fear of negative evaluation and the moderating role of self-esteem. It has been shown that social comparison (Christoforou, 2015), self-objectification (Lim, 2015), and social discrimination (Adams et al., 2017) have a certain impact on appearance anxiety, so we will consider including the above factors in future studies to gradually improve the research model.

## Data availability statement

The original contributions presented in the study are included in the article/supplementary material, further inquiries can be directed to the corresponding author.

## Ethics statement

The studies involving humans were approved by the Academic Committee of the School of Journalism and Communication of Huaqiao University. The studies were conducted in accordance with the local legislation and institutional requirements. Written informed consent from the patients/participants or the participants’ legal guardians/next of kin was not required to participate in this study in accordance with the national legislation and the institutional requirements.

## Author contributions

ZZ: Investigation, Writing – review & editing, Conceptualization, Methodology, Project administration, Supervision. MZ: Writing – original draft, Data curation, Investigation, Methodology, Software.
